# Youth Attitudes Towards Their Future: the Role of Resources, Agency and Individualism in the UK

**DOI:** 10.1007/s43151-021-00061-5

**Published:** 2021-11-25

**Authors:** Avril Keating, Gabriella Melis

**Affiliations:** 1 Institute of Education, University College London, London, England; 2 University of Liverpool, Liverpool, England

**Keywords:** Youth, Young people, Optimism, Agency, Individualism

## Abstract

Young adults tend to be more optimistic about the future than older people, even during social and economic crises such as those created by the COVID pandemic. In this paper, we analyse survey data from a previous economic crisis to examine why young adults remain optimistic about their personal futures, and to consider what lessons, if any, this can help us with thinking about a post-COVID future. The data in question are drawn from a unique cross-sectional survey of young adults aged 22–29 in England, Scotland and Wales conducted in 2014, when youth unemployment in the UK was still extraordinarily high. Using these data, we assess the effect of resources, agency and individualism on young adults’ optimism. Multiple regression models of these data show that individual resources and individual attitudes not only have an independent effect on levels of youth optimism, but they can also interact. In particular, we argue that self-efficacy is the strongest predictor of youth optimism, together with educational resources, but we also show that some youth attitudes (namely individualism) affect youth optimism in different ways, depending on the level of individual-level resources available to the young person. These findings highlight the complexity of understanding youth optimism and point us towards possibilities for supporting young adults in post-pandemic times.

## Introduction

As the consequences of the COVID-19 pandemic unfold, recent headlines about young people have included stark warnings about the future prospects of young adults. Although younger people are relatively protected from the physical consequences of the virus, it seems likely that young adults will bear the brunt of the economic, educational and social fallout from the COVID-19 pandemic, much like in previous recessions (Schoon and Mortimer 2017; Furlong et al. 2018). Despite the challenging context, we know from past crises that even in the face of great uncertainty, young adults tend to remain optimistic about their future prospects, particularly when compared with older age groups’ views of the future (Franceschelli and Keating 2018). Although surveys have frequently highlighted this pattern, there are conflicting explanations of why young adults are optimistic about the future. In this paper, therefore, we analyse survey data from a previous economic crisis to examine why young adults remain optimistic, and to consider what insights, if any, this can provide to help us with thinking about a post-COVID future. 

The data in question are drawn from a unique cross-sectional survey of young adults aged 22–29 in England, Scotland and Wales, conducted in 2014 (when youth employment prospects were still suffering the consequences of the 2008 Global Economic Crash^1^; see ONS 2014). Multiple regression of these data show that individual resources and individual agency not only have an independent effect on individual levels of youth optimism, but they also interact. These analyses shed further light on the complex relationships between youth attitudes, resources and optimism, and show that the relationship between youth attitudes and optimism varies depending on the level of resources available to the young adult. Those with scarcer resources, such as the NEET youth in our study, are more pessimistic than their peers only if they are highly individualistic, indicating an exacerbating effect of negative attitudes on a socio-economic disadvantaged position. These findings highlight the complexity of understanding youth optimism and point us towards possibilities for supporting young adults in post-pandemic times.

## Understanding Optimism and Youth Futures

International comparative surveys have repeatedly found that young adults are more optimistic about the future than their elders (see Ipsos 2018; Stellinger and Wintrebert 2008). The intergenerational differences are apparent in attitudes towards the future of their country and the world, as well as towards their own personal prospects (Ipsos 2018). In most cases, youth optimism about their *own* future tends to be higher than their optimism about the future of the country or the world. Our focus in this paper is on youth optimism towards their own personal prospects, here conceptualised as a generalised positive and hopeful belief that the future they aspire to can be achieved. We will not focus on what the exact nature of their aspirations is, nor their expectations of whether these specific aspirations are realisable. Aspirations are also idealistic projections about one’s future, but differ from the disposition of being optimistic because, in the academic literature anyway, aspirations are typically related to a specific outcome in the future (e.g. becoming a doctor or attending university) (see Khattab 2015). Expectations, on the other hand, convey what respondents believe will *actually* happen in the future as regard to their aspiration, as opposed to their *hope* of what will happen. Expectations are less idealistic and more rooted in the empirical reality of respondents’ experiences (see ibid). All three of these concepts are related but nonetheless distinct.

If we were to believe the predominant narratives about Millennials in the media, we might attribute the positivity of youth to their so-called sense of entitlement. Although newspapers frequently highlight that contemporary youth are ‘doomed’ because they have limited prospects, these same newspapers simultaneously perpetuate the idea that the latest generations are also ‘special snowflakes’ that have been raised with a sense of entitlement and expect the future to be handed to them on a plate (see also Twenge 2014). In short, according to this narrative, young adults are optimistic about the future because they believe they are entitled to achieve their aspirations, regardless of the socio-economic or individual circumstances they find themselves in. Alternatively, this hopeful perspective could be viewed as an indicator of youth ignorance, naivety or disregard for current and future challenges. Yet, this interpretation is difficult to sustain when numerous studies have shown that young adults are deeply concerned about a wide range of issues, including climate change, terrorism and economic uncertainty (Ipsos 2018; Black and Walsh 2019; Threadgold 2012; Cook 2016).

Diverse explanations are also apparent in the academic debate. In the field of psychology, for example, youth optimism is conceptualised as a positive force and a psychological resource that enables individuals to cope with the stress and uncertainty of this phase of the life course (Arnett et al. 2014: 572). According to Arnett (2014), early adulthood is no longer a short stepping stone between childhood and ‘full’ adulthood, but a distinct and drawn-out life phase that is filled with uncertainty and change. Optimism is thus one of the non-cognitive resources that emerging adults can draw on to navigate the challenges that they face during this phase. This psychological support function may explain why youth optimism remains high during economic crises (Arnett et al. 2014). By contrast, some sociologists in the UK have suggested that it could be a *lack* of stress that explains the high levels of optimism they found among UK youth (Furlong et al. 2018). According to this perspective, the uncertainty of young adulthood is now so pervasive that it is ‘baked in’ to how many contemporary youth view their experiences during this life stage. For example, they argue that young adults now expect to experience a period of precarious work, and this is taken for granted rather than a source of great stress. One reason for this, they suggest, is that this generation is less likely to see ‘traditional’ work as central to their identity and to place more value on job flexibility and the resultant time this affords for spending time with family, friends and on non-employment-related pursuits (ibid: 91). However, they also posit that it is not (just) that attitudes towards work have changed, but also that young graduates often assume that the precarity will be temporary, which is why they remain optimistic (ibid: 94).

Yet neither Arnett (2014) nor Furlong et al.’s (2018) explanations fully explain this phenomenon. In this paper, therefore, we have returned to the more traditional theoretical question in Youth Studies: what roles do structure and agency play?

### Agency

The relative importance of structure and agency is a persistent theme in sociological research on youth, and highly pertinent in this context. At first glance, youth perceptions of their own agency may be a more promising explanation for youth optimism. After all, the same surveys that found high levels of youth optimism (e.g. IPSOS 2018) also found young people are more likely to believe that they can have a positive impact on the world and make a positive difference to how their country is governed (i.e. high levels of self-efficacy, a key measure of individual agency; Schoon and Lyons-Amos 2017). Agency is ‘the capacity for meaningful and sustained action both within situations and across the life course’ and previous research has shown that there is a strong relationship between young people’s perceptions of their agency and their actual outcomes in adulthood (Hitlin and Johnson 2015: 1462). Such is the import of this disposition that positive perceptions of one’s agency can help some individuals overcome structural constraints such as social class (although there are limits to its transformative power; see Schoon and Heckhausen 2019). Indeed, Leccardi (2017: 349) argues that the very ‘action of viewing the future as a realm of possibility has the power to unlock our ability to take on the material constraints of the present’. Recent qualitative research in England provides some further insight into the mechanisms that facilitate the transformative potential of youth optimism (see Franceschelli and Keating 2018). This study argues that there is a widespread and deep-seated faith among young adults that ‘If I work hard enough I can achieve everything I want to achieve’*.* At the individual level, this faith in the power of hard work offers a sense of control (I can change my own circumstances) and a plan of action (by working hard) that provides reassurance and generates or sustains feelings of optimism. That said, we should also acknowledge that while planning for the future can provide reassurance for some young adults, for others, thinking about the future can be a source of anxiety, particularly as they are so aware of the uncertainty of contemporary life, especially when it comes to employment opportunities and working conditions (Alexander et al. 2020).

### The Future as Ideology and the Individualisation of Hope

One can also link youth optimism to the wider academic discussions about individualisation and neoliberalism that have been re-shaping Britain (see Franceschelli and Keating 2018). Put simply, individualisation theories suggest that the locus of control over future planning has shifted in late modern societies; in this context, young adults no longer have to follow the ‘standard biography’ but instead get to construct their own ‘choice biography’. This shift creates new opportunities (and risks) for young adults but also emphasises the role of individual agency in forging their own futures (see Beck 1992; and the debate between Woodman (2009, 2010) and Roberts (2010) about the role and limitations of Beck’s theories in understanding young lives in the contemporary context). Neoliberalism, meanwhile, perpetuates individualism both through its insistence that individuals are responsible for their own success or failures, and that the state should have a reduced role solving social problems. According to Black and Walsh (2019: 149), the individualising and responsibilising dictates of neoliberalism shape not just young people’s aspirations for the future, but also their perceptions for whether their aspirations are achievable. As a result, hope itself has been shaped by neoliberal discourses and has become positioned as an ideal characteristic of the neoliberal subjectivity. Neoliberalism not only implies that individuals control their own fate, but also promotes the idea that the free market offers a fair system where anyone who is talented and works hard can overcome all obstacles and achieve greater success (Brown et al. 2010). From a sociological perspective, then, an individual’s belief that ‘If I work hard enough I can achieve everything I want to achieve’ can be seen not just as a personal disposition but also a manifestation of a neoliberal meritocracy discourse that has become pervasive (see Littler 2017). Moreover, while optimism about the future can help young adults to achieve their much hoped for outcomes, in neoliberal societies, it can also obscure the obstacles placed in their way by social structures such as social class and racial and ethnic categorisations (Franceschelli and Keating 2018).

### The Role of Resources

This latter argument raises a further question: what is the relationship between social structures and young adults’ optimism about the future? To date, there has been little research on the relationship between optimism and social structures such as social class, and the evidence that does exist is not conclusive (and, if quantitative, often not based on advanced analysis).^2^ On the one hand, Arnett et al. (2014) found that young adults in the USA continued to be positive about their personal futures irrespective of their social class of origins and despite the economic fallout from the 2008 Global Economic Crisis. Arnett et al.’s (2014) suggestion implies that this is a rational coping strategy that young adults develop, and one which helps prevent other life-inhibiting problems (e.g. depression). This also implies that optimism is a strategy that is available to all, not just the better off. In Italy, by contrast, optimism is often a privilege reserved for young adults with enough economic and social capital to withstand the uncertainty of the present and have hope for the future (Leccardi 2017). Cross-national differences in cultural and institutional factors (such welfare systems and transition regimes) also contribute to explain the variable role of social class in these cases (ibid; Stellinger and Wintrebert 2008).

In the UK context, previous research suggests that family and individual-level resources are likely to play a key role in shaping youth optimism. According to Anderson et al. (2002: Sect. 83.5) ‘… access to resources has rather little impact on ambitions, but… [it] has a marked impact on confidence that plans will succeed and on the realism of plans.’ Yet, recent research suggests that there is a more complex relationship between youth optimism and individual- and family-level resources (see Franceschelli and Keating 2018). This study found that young adults across all social classes can be optimistic, but the function that it plays in young lives is quite distinct. When young adults have higher levels of educational and socio-economic resources (their own or their parents), then the positive attitudes that they have about the future are in keeping with what they have been told about how the world works and, moreover, in keeping with what are the likely outcomes; their higher levels of resources mean that they will, more often than not, achieve their aspirations. By contrast, for those with fewer resources and poorer prospects, optimism serves as an ‘anchor of hope’ that helps them cope with (and make sense of) the contradictions between the (neoliberal) messages they receive (*if you just work hard enough you will attain your goals*!) and the limited opportunities they see around themselves. This echoes the distinction that Wiles et al. (2008) made between ‘hope as want’ and ‘hope as expectation’ as they analysed terminal cancer patients’ attitudes towards the future. Optimism thus ‘represents a general desire for a positive future, rather than the expectation of a specific outcome…[and] the notion of “hope as want” shines light upon the ambiguities and tensions inherent in the concept of hope’ (Cook 2016: 519).

### Hypotheses

In sum, there has been little empirical research on optimism thus far, and certainly very little that combines advanced statistical analysis with the sociology of youth and the sociology of futures.^3^ In light of this, and drawing on the literature discussed above, this paper seeks to use quantitative survey data to test three hypotheses. First, in line with the social structures literature, we examine whether ‘young adults are more likely to be optimistic about their future if they have higher levels of socio-economic, housing and/ or educational resources’ (H1). Second, and drawing on the agency and individualisation literature, we examine whether ‘young adults are more likely to be optimistic about their future if they have higher levels of agency and/or individualism’ (H2). Finally, we also examine how structure and agency interact; although structure and agency are discussed here as separate influences, in practice, these variables are inter-related (Schoon and Heckhausen 2019). Our third hypothesis, therefore is, that ‘agency and individualism affect youth optimism in different ways, depending on the level of individual-level resources available to the young adult’ (H3). The data and methods we use to test these hypotheses are discussed in the next section.

## Data and Methods

The data used here are drawn from a unique web survey that was conducted in late June/early July 2014 among 2025 young adults aged 22–29 in England, Scotland and Wales. Interviews were completed with 1003 people in England, 520 in Scotland and 502 in Wales. Successive weights were created to ensure that the data was nationally representative, and to account for the cross-national demographic differences, the weights were constructed in two stages and using rim weighting.^4^ Descriptively, our sample includes more young women than young men (57.6% versus 42.4% male), but it is broadly representative in terms of ethnicity, qualification levels, current occupation (student/working/NEET) and parental education.

The survey was designed to assess factors affecting civic attitudes and engagement during early adulthood, and it includes items able to measure a wide range of attitudes and behaviours theoretically related to youth optimism, such as self-efficacy, importance of hard work, individualism as well as variables measuring personal socio-economic resources and those of the family of origin. Table 1 reports the population post-stratified weighted proportions for the socio-demographic variables (i.e. those for the controls and resources) used in our study.

**Table 1:** Descriptive statistics for the available cases

Controls	*N*	Weighted proportion
*Gender*: female (ref.: male)	2025	0.49
*Ethnicity*: other ethnic group (ref.: White British)	1987	0.22
Resources		
*Books at home*:	1801	
None/very few		0.23
Some		0.45
Many		0.32
*Father’s occupation*:	1624	
Unskilled/low skilled		0.43
Skilled		0.22
Professional		0.35
*Qualifications*:	1956	
Lower secondary and below		0.31
Upper secondary		0.22
Degree		0.47
*Occupation*:	1994	
Working		0.73
In education		0.1
NEET		0.17
*Accommodation status*	1969	
Living with parents		0.25
Independent living		0.75

CELS-CAWI 2014

The survey also included five items designed to measure levels of youth optimism about their personal future. From these items, we wanted to create a statistically robust and valid latent uni-dimensional construct (or factor) that would reduce the measurement error that derives from the crude use of observed indicators. Exploratory factor analysis (EFA) followed by confirmatory factor analysis (CFA) indicated that a three-item factor (which we labelled Youth Optimism) was the best fit—i.e. it was the factor with the highest log-likelihood values and the lowest Akaike information criterion (AIC) and the Bayesian information criterion (BIC) values^5^ (Schwarz 1978). The pattern of responses and wording of these three items are described in Table 2. For the CFA, we used models and estimation procedures for polytomous items (Ostini and Nering 2010), as offered within the item response theory (IRT) models approach and further elaborated within the generalised latent variable models (GLVMs) framework (Muthén 1983).

**Table 2:** Frequency distribution for the five items measuring young adults’ optimism, % values over valid cases

Optimism item	Strongly disagree	Disagree	Neither agree nor disagree	Agree	Strongly agree	Total *N* (%)
1) I am optimistic that there will be plenty of opportunities for me in life	4	14	26	45	11	1936 (100)
2) I expect to end up in a better job than my parents had	4	17	31	35	13	1912 (100)
3) It is easier now for people like me to improve things for themselves than it was for my parents	7	21	32	33	7	1919 (100)

CELS-CAWI 2014

We then used these data to run a series of stepwise multivariable regression models that would test our hypotheses. In the multivariable regression model, youth optimism ${y}_{j}^{*}$ is predicted by the direct effects of the variables representing controls, resources and agency:

$${y}_{j}^{*}=\alpha +\beta {x}_{j}+{\varepsilon }_{j}$$

where $\beta$ is the regression parameter for the direct effect of exogenous covariates ${x}_{j}$ on the outcome ${y}_{j}^{*}$; $\alpha$ is the model intercept; and $\varepsilon$ is the error term. The models are progressively more complex in terms of number of explanatory variables. Model 1 is the simplest model, and it includes our resource measures, as well as controls for gender and ethnicity. The analysis does not include controls for respondents’ specific aspirations for the future or their expectations as to whether these aspirations were achievable; unfortunately, data of this nature were not available in this data set. As noted above, we believe that generalised optimism about one’s future is distinct from aspirations and expectations; however, we were not able to take this into account in this analysis. Likewise, we are also unable to include controls for community-level resources or state-provided opportunity structures, both of which have also been found to shape youth transitions (see Schoon and Heckhausen 2019).

Model 1 considered a number of different individual-level resource types, namely cultural capital (measured by the number of books in the home); parental socio-economic status (measured by father’s occupation); the highest qualification achieved (HQA) by our young adult respondent; their current occupation (working/in education/or not in education or training (NEET); and their accommodation status (comparing living independently with living with parents). Given the impact of socio-economic status on life choices and life chances (Schoon and Heckhausen 2019), one might expect that young adults will have higher levels of optimism if they also have higher levels of socio-economic resources (e.g. cultural capital, parental SES). Likewise, we might also expect educational qualifications to play an important role, as young people with higher qualifications are more likely to earn more over the life course and have higher levels of civic engagement, health and wellbeing (see Green and Henseke 2016, 2017). Students and graduates are aware of this, and this knowledge likely contributes to their optimism (Hitlin and Johnson 2015). Finally, accommodation status has been included because living independently from one’s parents is an important marker of adulthood but it is a resource that is not available to all young adults because of the increasing cost of housing (Coulter et al. 2020; Green 2017).

To test Hypothesis 2, we included three items as indicators of agency and individualism in Model 2, namely:

1. Perceptions of importance of hard work (Hard Work), measured by the question ‘Please tell me how important you think it is for getting ahead in life hard work?’ The item was coded as a 4-point Likert type, with 1 = not important at all and 4 = essential.
2. Self-efficacy (Efficacy) that is the young person’s self-perception of having influence on wider society, which in this survey was captured via relationships to political institutions. The original question asked how much the young person agreed or disagreed with the statement: ‘People like me can have a real influence on government if they get involved’, on a 5-point Likert scale with 1 = strongly disagree and 5 = strongly agree. Despite its ostensible focus on government, political efficacy can be seen as a type of self-efficacy, as the previous literature has shown how the two concepts are closely linked, both theoretically and empirically (see Levy 2013; Morrell 2005; Yeich and Levine 1994).
3. Extreme individualism (Individualism). Again, on a 5-point Likert scale, the interviewee was questioned on the level of agreement with the statement ‘People should look out for themselves, not for other people’, with 1 = strongly disagree and 5 = strongly agree.

Based on the agency and individualism literature discussed above, we expect young adults with higher levels of these attitudes to report higher levels of optimism. Table 3 shows the frequency distribution in percentage values of the three attitude items.

**Table 3:** Frequency distribution for the three attitude items: hard work, efficacy and individualism

Hard work %		Efficacy %		Individualism %	
Not important	3	Strongly disagree	10	Strongly disagree	9
Not very important	8	Disagree	23	Disagree	35
Very important	45	Neither agree nor disagree	31	Neither agree nor disagree	33
Essential	44	Agree	30	Agree	17
		Strongly agree	6	Strongly agree	6
**Total *n***	**1944**		**1943**		**1972**

For the final model (Model 3), we tested interactions between our resources and agency variables to examine whether agency and individualism affect youth optimism in different ways, depending on the level of resources available to the young person (H3). In this model, we focused on interactions with the resources the individual has acquired during early adulthood (HQA, employment and housing status) rather than their parents’ resources; the results of Models 1 and 2 indicated that father’s occupation was not related to our outcome of interest (see Table 4). Here, we expect that the relationship between youth attitudes and youth optimism will vary according to the level of resources available to them.

**Table 4:** Regression models, standardised estimates, standard errors and *R*-squared value

Predictors	Model 1	Model 2	Model 3
	Resources	Attitudes	Attitudes*YP qualification, occupation, tenure
	Coefficient (SE)	Coefficient (SE)	Coefficient (SE)
YP Gender: female	− 0.0714 (0.0438)	− 0.0779 (0.0422)	− 0.0766 (0.0421)
YP Ethnicity: other ethnic group (ref: White British)	0.143** (0.0567)	0.115** (0.0547)	0.126** (0.0547)
Parents: some books (ref: very few)	0.0388 (0.0645)	0.0514 (0.0622)	0.0528 (0.0618)
Parents: many books	− 0.184*** (0.0707)	− 0.151** (0.0691)	− 0.150** (0.0688)
Father: occupation skilled (ref: unskilled)	− 0.0318 (0.0605)	− 0.0265 (0.0578)	− 0.0321 (0.0575)
Father: occupation professional	− 0.0323 (0.065)	− 0.0365 (0.0606)	− 0.0458 (0.0603)
YP qualification: upper secondary (ref: none to lower secondary)	0.0361 (0.0651)	0.0189 (0.0628)	0.00696 (0.399)
YP qualification: degree	0.241*** (0.0631)	0.201*** (0.0611)	0.0495 (0.356)
YP occupation: in education (ref: in work)	0.148** (0.0647)	0.144** (0.062)	− 0.402 (0.393)
YP occupation: NEET	− 0.208*** (0.0614)	− 0.215*** (0.059)	0.087 (0.385)
YP tenure: independent living (ref: living with parents)	0.214*** (0.05)	0.214*** (0.048)	− 0.314 (0.332)
YP attitudes (low to high): efficacy		0.191*** (0.019)	0.183*** (0.0609)
YP attitudes (low to high): individualism		0.114*** (0.0208)	0.0534 (0.0636)
YP attitude (not important to very important): hard work		0.247*** (0.029)	0.136 (0.0897)
**Interactions**			
Upper secondary*Efficacy			− 0.0016 (0.0581)
Degree*Efficacy			− 0.014 (0.0546)
In education*Efficacy			− 0.0609 (0.0576)
NEET*Efficacy			0.0316 (0.0533)
Upper secondary*Individualism			− 0.0633 (0.0609)
Degree*Individualism			− 0.0884 (0.0561)
In education*Individualism			0.166** (0.0665)
NEET*Individualism			− 0.164*** (0.0565)
Upper secondary*Hard work			0.0637 (0.0868)
Degree*Hard work			0.138 (0.0762)
In education*Hard work			0.0984 (0.0827)
NEET*Hard work			0.0192 (0.0891)
Independent living*Efficacy			0.0215 (0.0463)
Independent living*Individualism			0.170*** (0.0498)
Independent living*Hard work			0.0032 (0.0696)
Constant	− 0.197** (0.0817)	− 1.877*** (0.152)	− 1.356*** (0.412)
**Observations**	**2,025**	**2,025**	**2,025**
***R*-squared**	**0.05**	**0.136**	**0.153**

***p < 0.01, **p < 0.05

Finally, due to item non-response on key variables for our analysis, multiple imputation (MI) was applied (Rubin 1976). The main reason to impute missing data is to keep statistical power and reduce bias due to missingness (Pampaka et al. 2016). Multiple imputation is a particular imputation method that incorporates estimates of the random variation across results obtained from a number of imputed data sets, in an iterative process described by Rubin (Rubin 1987) and based on a specified imputation model. Plausible values for the missing data are created through a Bayesian estimation method and a Markov-Chain-Monte-Carlo (MCMC) simulation (Nielsen 2003; Schafer and Graham 2002). In Stata 16, we used augmented chained regression models. In this work, the number of imputed data sets was 10, so that the imputation process is repeated ten times. The results obtained and reported here for the multivariate regression models are the average of the estimates from each imputed data set, each one made up of a total of 2025 cases. Distinct from the analytical models, however, the imputation model contains all the variables and interaction terms explained below, plus age and parents’ highest occupation^6^ in order to enhance the quality of information used by the imputation algorithm.

## Findings

Model 1 is the simplest model and focuses on *resource* variables. Model 2 then also includes the three attitude items (hard work, efficacy and individualism). The final model (Model 3) then adds interaction terms between attitudes and qualifications, attitudes and occupation and attitudes and housing tenure (see Table 4). Model 3 explains 15.3% of the variance in youth optimism, whereas Model 1, containing only resources, explains just about 5% of the same outcome.

In Model 1, we find that youth optimism is significantly higher (at least at the 95% confidence level) among young adults who reported having a degree, being in education, living independently and ethnicities other than White British. By contrast, optimism is lower for those who are not in education, employment or training (i.e. NEETS). The results of the socio-economic variables produce a more complex picture. First, parental occupation is not statistically significant and second, young adults with the highest levels of cultural capital (i.e. books in the home) have *lower* levels of youth optimism, which is the inverse of the relationship we expected. It is also notable that unlike some of the other resources, this negative relationship between high cultural capital and optimism is sustained in all three models, even after youth attitudes and resource interactions are taken into account. Together, these findings suggest that young adults are indeed more likely to be optimistic about their future if they have higher levels of educational and housing resources (H1) but our assumptions about the relationship with socio-economic resources was not always borne out.

The results of Model 2 are more straightforward and in keeping with our theoretical assumptions (see Table 4). When attitudes are introduced in Model 2, we see that the variance explained for youth optimism jumps to 13.6% and that each of our measures of efficacy, individualism and hard work have positive effects on levels of optimism among young adults. The resources relationships that were identified in Model 1 remain statistically significant in Model 2, with only small changes in the strength of relationships assessed in Model 1. These results support the thesis that both attitudes *and* resources are related to young adults’ optimism (HP2).

It is in Model 3 that we see that youth attitudes and resources also interact, and that these attitudes affect youth optimism in different ways, depending on the level of resources that a young person has (as distinct from parental resources). For this model, we computed a series of interaction terms between the young adults resources and attitudes. This produced several notable results. First, this model confirms that there is an interaction between individualism and resources, and thus that youth levels of individualism enable us to distinguish between highly resourceful and more disadvantaged youth. More specifically, when compared with young adults who are working (the reference category in this instance), young adults that are NEET have significantly lower optimism if they are highly individualistic. Meanwhile, the opposite is true for their peers who are living outside of the family home, and/or who are in education or work; for young adults with these resources, the more individualistic they are, the more likely they are to be optimistic.

Second, the interaction between individualism and youth resources also has an impact on the other relationships in this model. In Model 3, youth optimism is no longer associated with having a degree, being in education, being NEET, living independently or reporting a strong belief in hard work; the main effects of these variables are no longer statistically significant. This indicates that when taken separately these resources and attitudes do not define youth optimism; instead, the interactions among these should be accounted for. On other hand, high levels of cultural capital still predict *lower* levels of optimism and self-efficacy continues to be statistically significant and the strongest predictor of youth optimism.

Finally, gender is not statistically significant in any of the models (cf: Webber and Smokowski 2018), but ethnicity is, with BAME youth consistently reporting higher levels of optimism in all three models. This is in line with previous research that shows that first- and second-generation immigrants have higher aspirations for, and greater optimism about, the future (see Cebolla-Boada et al. 2021); immigrant capital could thus be viewed as an additional ‘resource’ that some adults can draw on. The implications of these findings are discussed in the next section.

## Discussion and Conclusions

Our goal was to examine how agency and resources shape youth optimism about their personal futures, both separately and in interaction with one another. Previous research had indicated that various structural and attitudinal factors are associated with youth optimism, but few sociological studies have tested these theories using advanced statistical methods. This paper thus contributes to the field by using regression analyses and factor analysis to examine a wide range of variables and their relationship to each other and to youth optimism. Broadly speaking, our findings confirm expectations that individual-level resources and attitudes not only have an independent effect on levels of youth optimism, but they also interact. Yet, the analysis also showed that the relationship between resources, attitudes and optimism is more complex than we assumed. For one, contrary to expectations, those with the highest levels of cultural capital resources (books in the home) have *lower* rather than higher levels of optimism. One explanation for this could be that young adults with very high cultural capital are more aware of the challenges facing young adults in the current social, political and economic climate and that this awareness has, in turn, an impact on their optimism. They may also have greater awareness that this cohort of young adults is the first generation that is less likely to be as well off as their parents and less able to achieve the lifestyle that their parents had provided for them (see Green 2017).

Furthermore, the analysis also confirmed that some youth attitudes (namely individualism) affect youth optimism in different ways, depending on the level of individual-level resources available to the young person. More specifically, optimism is higher among the young adults who are still in education (rather than in work) *and* have higher levels of individualism. This relationship could be partly explained by their expectations about the rewards of higher qualifications (noted above), but also perhaps by the fact that competitive individualism has become such a prominent feature of the highly neoliberalised education culture in the UK (Keddie 2016; Brooks 2013). A similar pattern was found in relation to housing resources, although the underlying explanation may differ. Optimism is higher among young adults who are living independently and reported higher levels of individualism. In this case, it may be that this group is benefitting from a non-economic resource, namely accumulated experience of independent adulthood. This may give young adults more confidence that they will be able to achieve the future they aspire, as Arnett’s (2014) theory suggests.

By contrast, the interactions in Model 3 revealed that those who have low levels of resources (i.e. the NEETs) who are highly individualistic are *less* likely to be optimistic about their personal futures. This means that those with scarcer resources, such as the NEET youth in our study, are more pessimistic than their peers only if they are highly individualistic, indicating an exacerbating effect of individualistic attitudes on a socio-economic disadvantaged position. In other words, it is not simply the status of being NEET that lowers young adults’ optimism; after all, despite the negative stereotypes, there are a wide range of reasons young adults are in this category (see Mascherini 2016). Rather, it is being NEET *and* feeling that ‘people should look out for themselves, not for other people’ that predicts lower levels of optimism. Those who fall into this category may lack support from others, either from their personal networks or wider society, and feel that they have no option but to look out for themselves rather than others. According to Anderson et al. (2002), those with the fewest resources (or those with considerable family responsibilities) find it difficult to make any plans about the future, never mind feeling optimistic about their plans. For young adults in this position, the lack of resources or excess of responsibilities mean that they feel:

…largely stuck, at least at present, where they are. At best they may develop approaches to life designed to stave off even worse disasters or to provide some possible relief from their current straits. (Ibid: Section 9.6)

To put it another way, it would seem that ‘hope as want’ is not even an option for this sub-group, and that planning for a positive future is a luxury they cannot afford. This absence of hope is important because, using longitudinal survey data, Hitlin and Johnson (2015) found that pessimism about one’s future life prospects during youth can have a lasting impact. Pessimism during adolescence was associated with lower earnings, poorer health and wellbeing and a greater chance of having financial problems in later life. As those who are NEET and highly individualistic are more pessimistic, this group are missing out on the transformative effect on life chances that optimism provides for others (see Schoon and Heckhausen 2019).

What are the implications of these findings as we try to support young adults to forge positive and independent futures in a post-pandemic context? The lessons we can draw from these data are necessarily limited, as the data predates the pandemic and cannot take into account the unique circumstances of the current crisis (such as the impact of social isolation, or the safety net provided by furlough schemes). There are nonetheless some parallels (such as high rates of youth unemployment) that mean these findings can still contribute to the contemporary debate about possibilities and policies for youth futures. For instance, youth mental health was a concern for policymakers and youth NGOs even before the pandemic, but the impact of COVID-19 has exacerbated the crisis not just because of its impact on young people’s social lives, but also its impact on economic and educational futures (Henderson et al. 2020; Green et al. 2021). Optimism about the future (or lack thereof) can play a key role in mental health and employment outcomes (Hitlin and Johnson 2015), and thus, it is vital that governments invest in policies and programmes that provide young people with a sense of optimism that the future they aspire to is still attainable. The findings suggest that one way to do this is to increase opportunities for education and independent (and affordable) housing—i.e. resources that enable young people to become independent adults. Yet, it is not just a matter of resources: in these analyses, self-efficacy was the strongest predictor of youth optimism, suggesting that including young adults in decision-making, and providing them with a sense that they can influence the world around them, can boost their optimism about their future and, in turn, contribute long term to facilitating positive outcomes in later adulthood. While all young people can benefit, these types of tools and opportunities are particularly important for NEETS that lack economic resources and strong social networks; this group is most vulnerable to the long-term scarring effects of COVID-related curtailments. As sociologists concerned about youth futures, we remain optimistic that young adults can still achieve their ambitions in this challenging socio-economic environment, if they receive the right support from governments and society. After all, as Cook and Cuervo (2019) point out, hope is not static; rather, it changes over time in response to shifting circumstances, social relations and subjectivities.

## Footnotes

1 At the end of 2013, the youth unemployment in Britain was 20%, just two percentage points lower than the peak of post-GEC youth unemployment in 2011 (ONS 2014: 6).

2 Most of the quantitative analysis that does exist seems to be based solely on descriptive statistics and basic cross-tabs. Notable exceptions to this are Simões et al.’s (2021) and Hitlin and Johnson’s (2015) paper using longitudinal survey data.

3 Hitlin and Johnson’s (2015) quantitative analysis tests social psychology theories.

4 First, data for each country (England, Scotland and Wales) was weighted to ensure that it was nationally representative of young adults living in each country in terms of gender, region, ethnicity and highest qualification. Second, weights were then applied to achieve the correct proportion for three countries in relation to each other.

5 The full results and all online supplementary material are available on Figshare at this address: 10.6084/m9.figshare.17049245.v2.

6 The imputation model includes both fathers’ and both parents’ education to account for mothers’ education when available but still retain all info for father, and/or to account for mothers’ education when this is the highest qualification achieved in the household.
